# omniCLIP: probabilistic identification of protein-RNA interactions from CLIP-seq data

**DOI:** 10.1186/s13059-018-1521-2

**Published:** 2018-11-01

**Authors:** Philipp Drewe-Boss, Hans-Hermann Wessels, Uwe Ohler

**Affiliations:** 1 Berlin Institute for Medical Systems Biology, Max Delbrück Center for Molecular Medicine in the Helmholtz Association, Robert-Rössle-Strasse 10, Berlin, 13125 Germany; 2 Department of Biology, Humboldt University, Berlin, Germany

**Keywords:** Machine learning, Bioinformatics, Protein-RNA interactions, CLIP-seq, eCLIP, iCLIP, PAR-CLIP, HITS-CLIP, Generalized linear models, Mixture models

## Abstract

CLIP-seq methods allow the generation of genome-wide maps of RNA binding protein – RNA interaction sites. However, due to differences between different CLIP-seq assays, existing computational approaches to analyze the data can only be applied to a subset of assays. Here, we present a probabilistic model called omniCLIP that can detect regulatory elements in RNAs from data of all CLIP-seq assays. omniCLIP jointly models data across replicates and can integrate background information. Therefore, omniCLIP greatly simplifies the data analysis, increases the reliability of results and paves the way for integrative studies based on data from different assays.

**Electronic supplementary material:**

The online version of this article (10.1186/s13059-018-1521-2) contains supplementary material, which is available to authorized users.

## Background

All RNA molecules are subject to post-transcriptional gene regulation (PTGR) mechanisms, including sequence-, structure- and RNA-modification-dependent modulation of splicing, cleavage and polyadenylation, editing, transport, stability, and translation. In the regulation of PTGR RNA-binding proteins (RBPs) play an important role. Many RBPs are required for constitutive processes, such as pre-mRNA splicing, cleavage, and polyadenylation. Furthermore, cell-type specific RBPs and non-coding RNAs can regulate the flow of genetic information in more directed manners, e.g. by regulating mRNA stability or translation. The complex orchestration of RBPs upon their respective targets ultimately determines appropriate protein expression.

The complexity and importance of PTGR is underscored by the large number of RBPs that have been identified in recent genomics and proteomics studies [1], as well as the wide range of diseases that result from genetic alterations within RBPs and/or their mRNA targets [2, 3]. Despite this large number of human RBPs, for the vast majority, neither their targets nor functions are well understood. Uncovering the regulatory sequence elements and important RNA-RBP interactions will be critical to interpret human genetic variation in regulatory RNA regions and in the noncoding transcripts that are increasingly uncovered by genome-wide deep sequencing [4, 5].

Deep sequencing technologies have enabled the development of various new protocols for mapping interaction sites between RBPs and their RNA target sites, as well as for identifying RNA-modifications on a genome-wide scale. Therefore, it is now possible to resolve interdependencies and redundancies of binding of RBPs and ribonucleoprotein particles (RNPs) to mRNA molecules and evaluate the contribution of these interactions to gene regulation in the context of cellular metabolism, organismal development or normal and disease states [6, 7]. Experimental approaches to study genome-wide RNA-RBP interactions include different variants of cross-linking and immunoprecipitation (CLIP) protocols: high-throughput sequencing of RNA isolated by crosslinking immunoprecipitation (HITS-CLIP) [8], photoactivatable ribonucleoside enhanced cross-linking and immunoprecipitation (PAR-CLIP) [9], individual nucleotide resolution cross-linking and immunoprecipitation (iCLIP) [10], individual-nucleotide resolution crosslinking and affinity purification (iCLAP) [11], crosslinking and cDNA analysis (CRAC) [12], enhanced CLIP (eCLIP) [13] and other methods. Similar principles have also motivated the development of protocols to study transcript modifications such as m6A-seq [14] or Pseudo-seq [15]. These protocols all have in common that they enable sequencing of RNA-fragments that were bound by a specific RBP or carry a modification, via antibodies against the native protein, modification or tagged transgenic RBPs.

Due to biochemical properties of RBP cross-linking, the resulting reads contain conversions, deletions or truncations at or near the cross-linked sites. These so-called diagnostic events are indicative of RNA-RBP interactions or RNA modifications and thus enable nucleotide-level identification of the binding sites. For PAR-CLIP the most common diagnostic event type is a T-C conversion, for iCLIP and eCLIP it is a truncation and for HITS-CLIP a deletion. It should be noted, however, that there can be also less abundant secondary diagnostic event types at the interaction sites [16].

Similar to ChIP-seq [17], the resulting data from these protocols exhibits pileups of reads (peaks) near interactions sites. The height of peaks is influenced by factors such as the strength of binding, interaction or competition with other RBPs, local biases induced by differences in RNAse digestion efficiencies and PCR-biases [18]. A fundamental difference to ChIP-seq, however, is that the coverage at interactions sites, but to a smaller degree also at non-binding sites, is strongly influenced by the wide magnitude of RNA expression levels, i.e. the relative abundance/availability of the transcript that was bound. The extend of confounding of the peak height by factors apart from the binding strength, can be estimated from input or background libraries, which include most steps of the CLIP protocols except the immunoprecipitation. Another challenge of the data is that there are often spurious peaks at locations that do not show the typical characteristics of binding sites (e.g. motifs). In summary, the challenge of CLIP data analysis includes the proper modelling of peak height and the diagnostic events, while accounting for confounding factors and modelling of technical and biological variance.

Various methods have been proposed to recover the interaction sites from sequencing data [19, 20]. PARalyzer [21], the first dedicated tool for PAR-CLIP data analysis, mapped sites via local maxima of kernel-smoothed profiles of T-C conversion events. WavCluster [22] models the T-C conversions and sequencing errors using a binomial distribution and estimates a background threshold to identify peak boundaries. The binomial model of T-C conversions is extended by BMIX [23]) to also model sequence variants. Methods that do not model the diagnostic events include Piranha [24], which determines bins of fixed size that have a higher number of read starts than expected by chance. Piranha was the first method to model the CLIP-reads using a Negative binomial distribution and principle also allows including covariates. Clipper [25] is another methods that does not uses diagnostic events. It models background read-counts using a Poisson distribution and identifies regions that are higher than expected by chance. However, all these methods suffer from at least one of the following shortcomings: (1) They do not contain an explicit model for diagnostic events or they can be only applied to a specific CLIP protocol as the modelling of diagnostic events is restricted to only one of the diagnostic event types. (2) They do not allow accounting for confounding factors, e.g. the gene expression. This can lead to a high false positive rate of peaks in highly expressed genes and at the same time a low true positive rate for peaks in lowly expressed genes. (3) As many early datasets did not provide background or input control libraries, many tools do not support integration of such data. Most tools also cannot handle replicate data and thus cannot account for biological variance, leading to poorly calibrated methods.

## Results

### A novel approach for identification of RBP-RNA interaction sites for all CLIP-seq assays

**Fig. 1:**
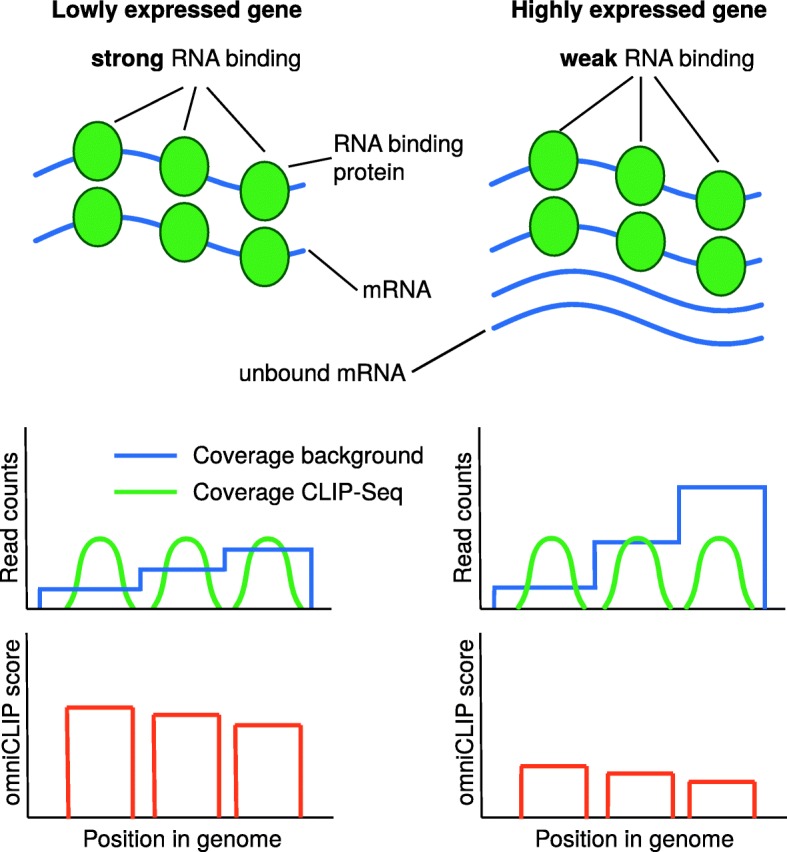
Illustration of omniCLIP peak calling. Shown on the (left) is peak calling for a lowly expressed genes with strong RNA binding protein (RBP) RNA-binding and on the (right) a highly expressed gene with weak RBP RNA-binding. During peak calling, local background coverage is taken into account to correct for confounding by local effects or biases. Furthermore, overall the RNA abundance is estimated and accounted for. This allows better ranking peaks with identical local coverage by sharing of information along the transcript, as illustrated for the leftmost peaks in both genes

**Fig. 2:**
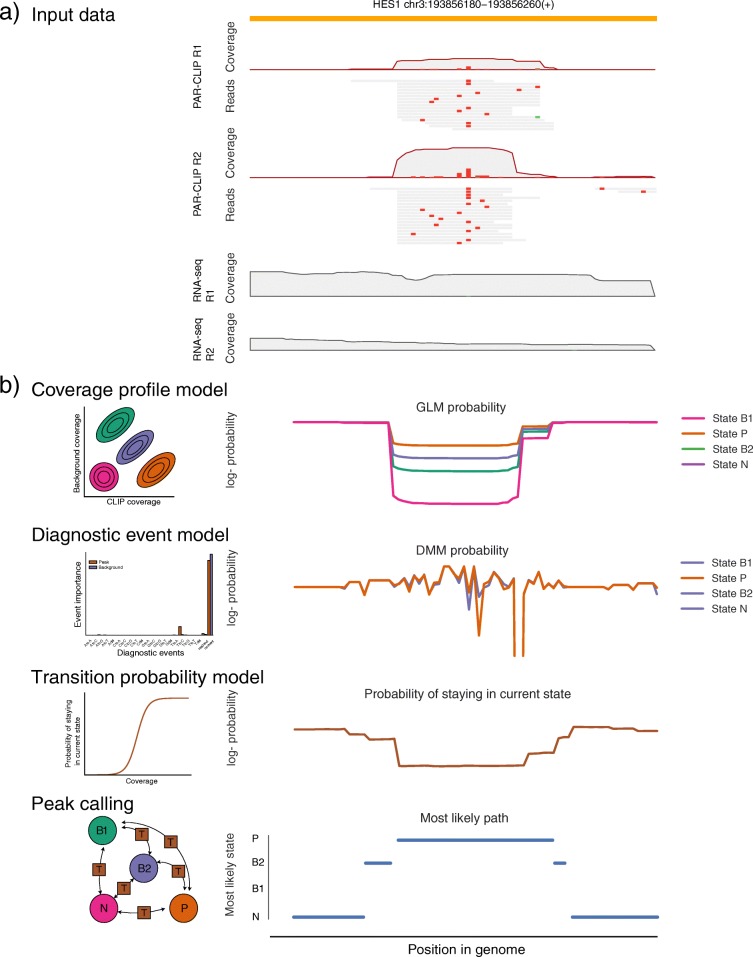
Illustration of omniCLIP application. **a** Shown is the read coverage of HES1 for two PAR-CLIP and two RNA-seq libraries as well as reads with diagnostic events. Here, the T-C conversions are shown in (red). **b** Application of omniCLIP. First, the probability of each position and each state is computed using the coverage profile model and the diagnostic event model. Next, the transition probabilities are computed based on the coverage at each position. Finally, a Non-homogeneous Hidden Markov Model is applied to segment the sequence in to peak regions (P) and non-peak regions (N, B1, B2)

To address the shortcomings of existing methods, we developed a new probabilistic method (omniCLIP) to identify regulatory regions from all of the aforementioned CLIP-seq protocols (see Fig. 1). The basic principle of our model is to identify target sites via an unsupervised segmentation of the genome. To this end, omniCLIP learns the relevant diagnostic events directly from the data and automatically uses them for peak calling. Furthermore, it explicitly accounts for confounding factors as well as technical and biological variance. To achieve this, we employ a Non-Homogeneous Hidden Markov Model (NHMM) to segment the genome into peaks and non-peaks. The emission probability of the NHMM is given by the product of the joint probability of the coverage and the probability of the observed diagnostic event frequency in all replicate CLIP and background libraries. To model coverage, we use a Negative Binomial based Generalized Linear Model (GLM) that models both confounding by the gene expression, confounding of local effects and also allows to account for excess variance. The diagnostic events are modeled using a Dirichlet-Multinomial mixture (DMM) model. The transition probabilities of the model are based on a logistic function that depends on the coverage. All parameters of the model are learned from the data, making it easily applicable to data from various protocols (see Fig. 2 for an illustration of the omniCLIP components and their application).

### Evaluation of omniCLIP on PAR-CLIP data

**Fig. 3:**
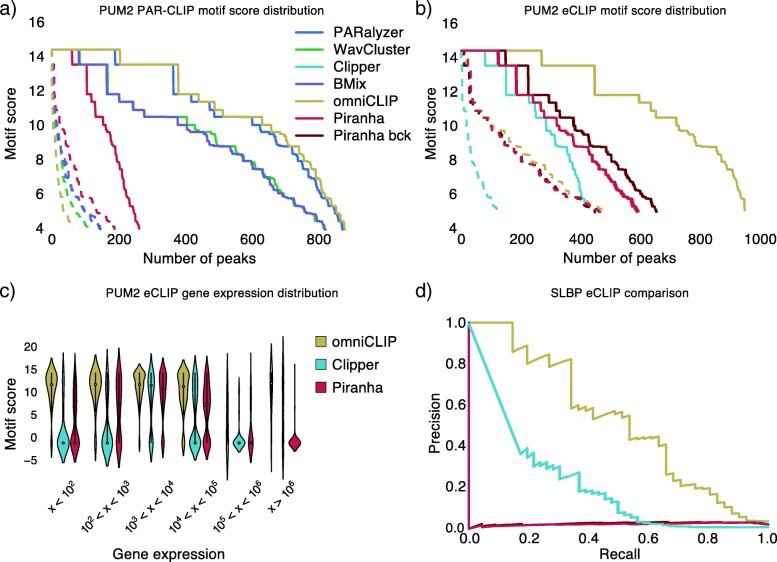
Performance evaluation. **a** Sorted distribution the PUM2 motif scores of the top 1,000 called peaks for PARalyzer, Piranha, WavCluster, BMix and omniCLIP on a HEK293 PUM2 PAR-CLIP dataset. Dashed lines indicate motif enrichments in a random control **b** PUM2 motif scores distribution for the top 1,000 called peaks for omniCLIP, Clipper and Piranha on a HepG2 PUM2 eCLIP dataset. **c** Motif score distribution for the top 1,000 peaks on the HepG2 PUM2 eCLIP dataset. Peaks are further classified by gene expression. **d** Precision recall curves for Clipper Piranha and omniCLIP on a HepG2 SLBP dataset for discriminating histone genes and non-histone genes from peak scores

To showcase the versatile abilities of omniCLIP, we demonstrate its application across data from different CLIP protocols, for RBPs that enable an independent evaluation of the quality of peak calls as well as on simulated data. First, we assessed its performance on PAR-CLIP [9] and eCLIP experiments for Pumilio 2 (PUM2), a RNA binding factor with a known high sequence specificity. To this end, we compared the predictions with those from other PAR-CLIP methods, including PARalyzer, WavCluster, BMIX and a general peak caller Piranha. On this PAR-CLIP dataset obtained from the human HEK293 cell line, omniCLIP and PARalyzer called the highest number of peaks (*n*=13,292 and *n*=5,602, respectively) followed by BMix (*n*=4,501), WavCluster (*n*=2,473) and Piranha (*n*=678). As there is no matching PAR-CLIP background dataset available for PUM2, we used two HEK293 ribo-zero RNA-seq libraries as background [7]. To evaluate the quality of the called peaks, we analyzed the enrichment of high-scoring PUM2 motifs in the peaks, which we take as indicators of high-affinity binding sites. As the number of peaks called by the different methods varied by an order of magnitude due to different cut-offs for peak calling, we compared the enrichment in the top 1,000 peaks of each method. For methods where no ranking criterion for peaks was provided, we used a random sub-selection of peaks (see Fig. 3a). The difference to the other methods, was especially strong for peaks that had a high motif score. All the enrichments are higher than expected by chance (see Fig. 3a).

We, furthermore, investigated how different random initialisations of our method affect the prediction. For this, we applied omniCLIP with 100 different random parameter initialisations. On average 98.6*%* of peaks overlapped between the runs, showing that omniCLIP fitting is robust to different initialisations.

### Evaluation of omniCLIP on eCLIP data

We then applied omniCLIP to a PUM2 eCLIP dataset from the human K562 cell lines that we obtained from ENCODE. Here, we compared omniCLIP with Clipper and Piranha. We applied Piranha with and without providing it the background as a covariate. As eCLIP peaks typically have longer tails than PAR-CLIP peaks, we used for omniCLIP the central high confidence-parts of the peaks. Applying Clipper results in on average 43,594 peaks per replicates, whereas omniCLIP found 21,654 peaks and Piranha 10,564 peaks, with omniCLIP exhibiting the highest enrichment of high scoring motifs in the top 1,000 peaks (see Fig. 3b). Again the enrichment of high scores in the top 1,000 peaks was not due to chance (see Fig. 3b). To analyse how gene expression influences the quality of the detected peaks, we binned the top 1,000 peaks based on the expression level of the gene, in which they were identified (see Fig. 3c). We found that for omniCLIP the top 1,000 peaks were in genes that had a lower expression than those genes in which the top 1,000 peaks of Clipper and Piranha were found. Furthermore, we found for Clipper and Piranha a strong dependence of the motif score of a peak and expression of the gene in which the peak was located. In peaks within genes with less than 10^4^ read counts, omniCLIP, Piranha and Clipper 84% (827 of 985), 52% (430 of 824) and 44% (346 of 792) contained high scoring motifs (*x*>8.0), respectively. This was very different for peaks in genes with more than 10,000 counts: Here, 50% (9 of 15), 6% (11 of 176) and 9% (18 of 208) of omniCLIP, Piranha and Clipper peaks had high scoring motifs. This suggests that omniCLIP has a better calibration than Clipper and Piranha, especially for highly expressed genes.

**Fig. 4:**
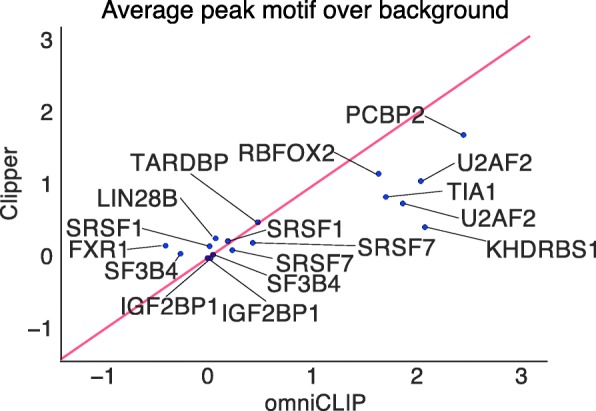
eCLIP analysis. Shown is the mean average motif score of the top 1,000 peaks over background for Clipper and omniCLIP for selected eCLIP experiments

We further applied Clipper and omniCLIP to all other eCLIP datasets for which motifs of length ≥6 from [26] were available. In total, we identified 12 RBPs with both eCLIP data as well as motifs (see Additional file 1: Supplemental Table S1). On this data, we applied Clipper and omniCLIP. For both methods, we determined for each RBP the difference between the average motif score of the top 1,000 peaks and the average motif score in a random control. We found that there were two classes of RBPs (see Fig. 4): Those for which the difference was small and those for which it was large. For the first class of RBPs the difference was comparable between Clipper and omniCLIP. For the class of RBP where the motif enrichment was stronger than expected by chance, however, omniCLIP performed better than Clipper. This suggests that when the motif-score is a good evaluation criterion for peak qualities, omniCLIP performs better than Clipper. The two different classes of RBP could be due to several biological or technical reasons: That for a subset of RBPs the motif alone is not sufficient to characterise the binding sites, that the motif does not reflect the *in-vivo* binding preference of the RBPs or that the CLIP-library qualities were poor.

Available eCLIP data for SLBP allowed for another independent validation of peak calls, as it is known to bind specifically the 3’-ends of histone-gene mRNAs. Thus, peaks in histone transcripts should have a higher score than those found in other transcripts. Therefore, we combined the scores of all peaks in a gene and measured via the area under the precision-recall curve (auPRC), how well the scores allow distinguishing of histone-genes from other genes. Here, omniCLIP achieved an auPRC of 0.52, Clipper an auPRC of 0.21, and Piranha an auPRC of 0.03 and 0.02 with and without using the background CLIP data, respectively (see Fig. 3d).

### Evaluation of omniCLIP on HITS-CLIP data

**Fig. 5:**
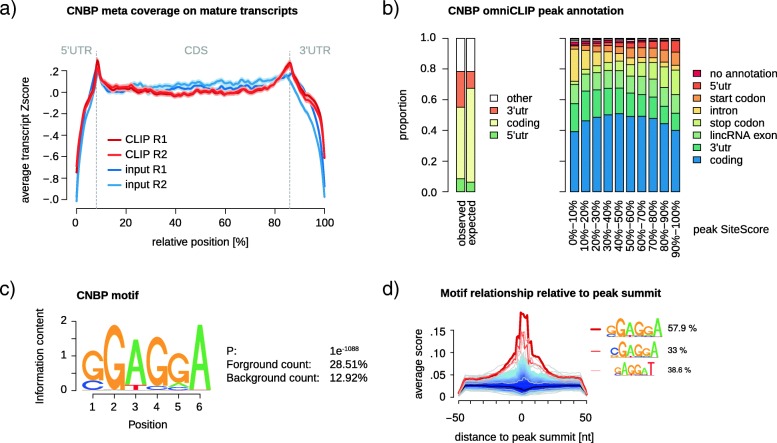
Binding preferences of CNBP. **a** Metaplot depicting the average Z-score transformed binned coverage across all genes (transcript with highest RSEM isoform percentage selected) with omniCLIP peak. Median 5’UTR (8%), CDS (78%) and 3’UTR (14%) proportions were extracted from all expressed genes in *Drosophila* S2 cells (TPM >0) from regular total RNA-seq experiments. Shades around solid lines indicate the standard error. **b** omniCLIP peak annotation grouped by strength into 10 peak SiteScore bins. (Left) Simplified annotation categories, to enable comparison to the expected annotation distribution. Here, 5’UTR contains the start codon and 3’UTR the stop codon, respectively. The expected peak annotation distribution was calculated according to the feature distribution shown in (**a**), for all peaks that are annotated as mature transcripts. Peaks classified as ‘other’ were ignored. (Right) Peak annotation categories grouped by peak score. Peaks annotated with start or stop codon do overlap such features. **c** CNBP motif calculated using HOMER2 for all peaks annotated to mature transcripts (*n*=29556), relative to 10x dinucleotide shuffled background sequences. **d** Recovery of the CNBP motif and shuffled PWM relative to peak summit of all peaks used (n = 29,433). PWM match required 80% similarity. Indicated percentages reflect peak sequences with motif hit. The next highest recovered random PWMs are variants of the identified motif

To demonstrate that omniCLIP can also be used to analyze HITS-CLIP data, we applied it on two libraries for the *Drosophila* RBP CNBP (CG3800), which we have previously identified as an unconventional RBP [27]. CNBP binds mainly to mature mRNA sequences in *Drosophila* and *human* [27, 28]. Within these sequences, CNBP shows a slight preference for binding of start and stop codon proximal regions, relative to input (see Fig. 5a) Both *Drosophila* CNBP HITS-CLIP replicates come with size matched UV-crosslinked input control of digested total RNA, collected prior to immunoprecipitation. Importantly, input RNA fragments undergo a library cloning procedure very similar to HITS-CLIP libraries, including RNA fragment size selection and adapter ligation, resulting in highly accurate backgrounds. Application of omniCLIP resulted in 34,224 peaks. The peaks show increasing annotation to start and stop codon categories with increasing peak scores (see Fig. 5b). This is in agreement with human CNBP, which was recently shown to bind preferentially to regions close to start codons [28]. We identified the highly significant GGAGGA motif relative to dinucleotide shuffled background (see Additional file 1: Supplemental Table S2) in omniCLIP peaks annotated to be mature mRNA sequences (see Fig. 5c). This confirms the reported k-mer enrichment relative to input in concurrent in vitro and in vivo studies of the human CNBP ortholog [28, 29]. Furthermore, we saw a strong connection of the motif residing in proximity to the peaks summit (see Fig. 5d), suggesting that omniCLIP can reliably resolve biologically relevant interaction sites in HITS-CLIP data, even with low frequencies of diagnostic events.

### Evaluation of omniCLIP on iCLIP data

**Fig. 6:**
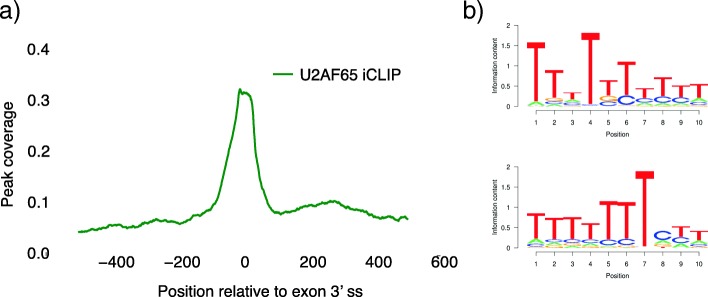
iCLIP U2AF65 analysis. **a** Shown is the average peak density around the 3’ splice site of exons from an iCLIP experiment of U2AF65. **b** Shows are the top two discovered motifs in the U2AF65-peaks

Finally, we applied omniCLIP to an iCLIP dataset for the splice factor U2AF65 [30]. The RBP U2AF65 is known to bind a polypyrimidine tracts motif 3’ of alternative exons splice sites [30, 31]. When applying omniCLIP we recovered both the reported binding preference and also the reported motifs (see Fig. 6). This shows that omniCLIP can also be applied to iCLIP data.

### Evaluation of omniCLIP on simulated data

**Fig. 7:**
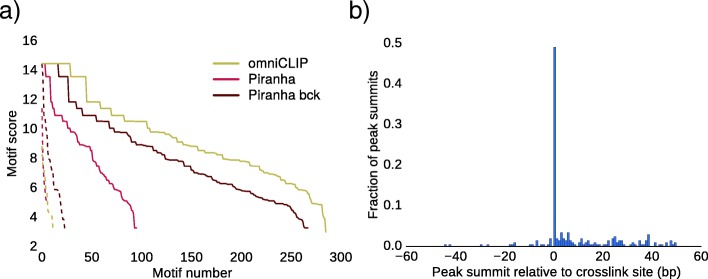
Simulated data analysis. **a** Shown is the sorted distribution of all PUM2 motif scores of the called peaks for omniCLIP and Piranha on a simulated CLIP-seq dataset. **b** Shown is the fraction of peaks summits relative to the simulated A-T crosslink-site in the PUM2-motif

To demonstrate that omniCLIP can also be applied when diagnostic events, other than T-C conversion, truncations or deletions, are present, we simulated CLIP-seq libraries with A-T conversions at the crosslinking-site. To this end, we simulated two biological replicates of PUM2 CLIP-seq libraries and two biological replicates of background libraries for chr1 of the *human* genome. We then induced A-T conversion at the fourth position of the PUM2 binding motif in the CLIP-seq libraries. On this dataset we first applied omniCLIP and Piranha (with and without background). Overall, omniCLIP found 333 peaks whereas Piranha when using the background found 323 and when not using the background found only 108 peaks (see Fig. 7a), showing that also on this dataset omniCLIP compares favourably against other methods. We further analysed the position of the site with the strongest diagnostic event score relative to the simulated cross-linking site. Here, we found that 49.2*%* of the peak summits were at the cross-linking site (see Fig. 7b). Finally, we compared the estimate GLM expression parameters with the simulated abundances per gene (see Additional file 1: Supplemental Figure S3). We found that the estimated gene expression correlated highly (Spearman *r*=0.82) with the ground truth expressions, showing that the parameter estimation is robust.

## Discussion and conclusions

Understanding the mechanisms of RNA-processing and their role in development or diseases requires understanding RBP-RNA interactions and functional consequences of these interactions. This depends on reliably identifying RBP-RNA interaction sites. However, determining the interaction sites from CLIP-seq data is challenging due to the presence of many confounding factors.

Here, we present omniCLIP, a probabilistic approach to identify regulatory elements from CLIP-data. Our model presents a principled framework for the analysis of RNA interaction assays and takes into account several important new aspects. First, we jointly model the observed coverage in all replicates. This allows for including replicate information and also accounting for various confounding factors. Additionally, we use an empirical Bayesian approach to identify and model important diagnostic events and sequencing errors. Finally, we take both biological and technical variance into account in our model. Overall, jointly modelling all information and uncertainties allows determining an accurate picture of the RNA-RBP interaction landscape.

We show that omniCLIP can be applied to data from a wide-range of CLIP-protocols, with superior performance to existing methods. This shows that it can be easily applied to new protocols, as all parameters were learned from the data. Consequently, omniCLIP greatly simplifies analysis of novel CLIP-seq assays. For instance, as CLIP-seq protocols are conceptually similar to RNA modification sequencing, omniCLIP should be easily applicable to identify RNA modifications.

Another advantage of omniCLIP is that it models the data in a principled way, i.e. each of its components has a clear probabilistic interpretation. This enables an easy integration of other probabilistic models in omniCLIP, such as for binding motif, structure, for various biases or explicit models of additional confounding factors.

In omniCLIP, the quantitative model of the read abundances plays a crucial role in peak calling. It works best if read-numbers in the foreground represent the number of transcripts that were bound by the RBP of interest. In cases where for example no random barcodes were used, PCR-duplicates cannot be resolved, which effectively leads to a higher variance of the peak heights (see Additional file 1: Supplemental Figure S1). This might explain why omniCLIP performs only slightly better than PARalyzer, which draws most of its strength from diagnostic events, on the PUM2 PAR-CLIP datasets, as the PUM2 PAR-CLIP libraries were generated without random barcodes.

But also the data used for the background modelling plays an important role in omniCLIP, as it is utilized to estimate confounding by gene expression and local biases. Furthermore, it is also used to calibrate the diagnostic event model. Therefore, we recommend using an input as a background dataset. Yet, in many, especially early published CLIP studies, this data was not acquired. In this situation, less specific data such as RNA-seq data can serve as a substitute to some extent, but local biases are not captured using this data (see Additional file 1: Supplemental Figure S2 for example) and also the diagnostic event model may be less accurate. In the case when a specific background or input dataset is not available, we recommend to trim reads prior, to alignment and to match CLIP-seq read lengths in order to increase the similarity to CLIP-data. To further minimize the technical variability of the data, we suggest using a high quality alignment. For this, we recommend to remove multi-mapping reads and to use a stringent cut-off on the number of mismatches [32]. We recommend using simulated data to guide parameter choice for read processing.

In summary, we have evaluated omniCLIP on various datasets for which either high-quality motifs are available or the target genes are known. In all of these scenarios, we show that the omniCLIP performance is at least comparable or better than each method that we have compared it against. This is insofar remarkable as most competitor methods are tuned for specific protocols, and underlines omniCLIP’s potential for integrative transcriptome studies on different CLIP-seq assays.

## Methods

**Fig. 8:**
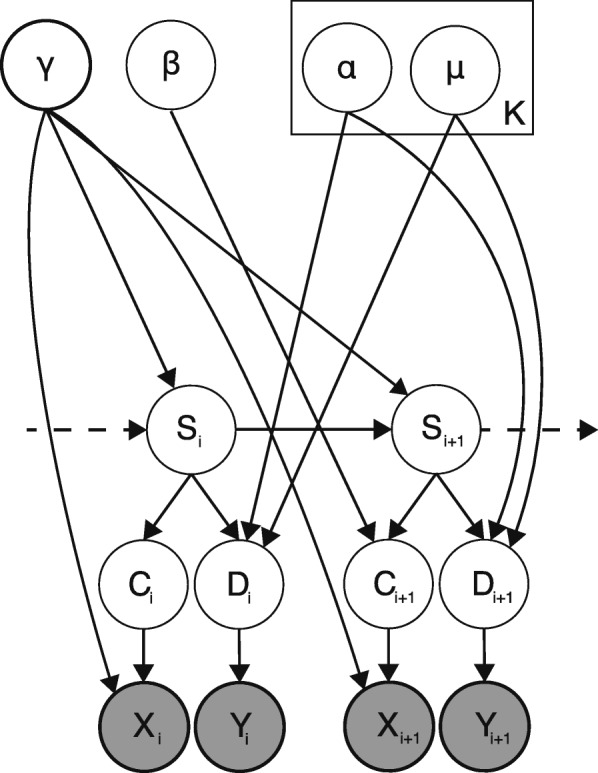
Plate diagram of omniCLIP model. Grey shaded elements indicate observed variable and unshaded elements indicate latent variables. Here, *X*_*i*_ and *Y*_*i*_ denote the observed coverage and diagnostic events at position *i* respectively, *C*_*i*_ the probability of the observed coverage, *D*_*i*_ the probability of the diagnostic events and *S*_*i*_ the state. Furthermore, *γ* denotes the transition probability and *β* the GLM parameters. Finally, *K* is the number of mixture components of the diagnostic event model, *μ* and *α* the multinomial Dirichlet mixture weights and parameters, respectively

***Model overview*** We model the observed reads in all libraries using a NHMM with four states: a state to model regions that are peaks (P), two states to model regions where the background signal is as high as the CLIP-seq signal (B2) or higher (B1) and a state that models regions with little or no coverage in any library (N). The emission probability of the NHMM is computed using a coverage profile model and a diagnostic event model. The transition probabilities depend of the local coverage. An overview of the model is shown in Fig 8.

***Modelling of the spatial dependence*** The transition probabilities between the states are modelled using a logistic function of the coverage *X*_*i*_ at position *i* in all replicates. This allows the model to be more or less rigid, depending on the amount of data that is available at a given position. Specifically, the probability *p*_*s*,*t*,*i*_ of a transition from state *s* to state *t* at position *i* is given by:

1 $$ p_{s,t,i} = \left\{\begin{array}{ll} f(X_{i}), &\text{if} s=t\\ \frac{1-f(X_{i})}{3}, & \text{otherwise} \end{array},\right.  $$

where *f* is a logistic function. Here, the probability of remaining in the same state is identical for all states. The probability of transitioning to any other state is uniformly distributed. The parameters of *f* are learned using stochastic gradient descent.

***Coverage profile model*** We jointly model the coverage in all replicates of the CLIP- and background-dataset. In our model, we assume that the coverage at each position of the genome follows a Negative Binomial (NB) distribution that is determined by the library size, the gene expression, and whether the position is a peak. We model this dependence using a generalized linear model (GLM) in the following manner. Assume that we have *U* CLIP and *V* background datasets and *G* genes. Then, we assume that the expected coverage $X^{u}_{i}$ in the CLIP-library *u* for each position *i* in a gene *g*∈{1,…,*G*} depending on the state *s* is determined by:

2 $$ \log(X^{u}_{i}) = \left\{\begin{array}{ll} l_{u} + \beta_{g} + \beta_{G+1}, &\text{if s}=P\\ l_{u} + \beta_{g} - \beta_{G+1}, &\text{if s}=B1\\ l_{u} + \beta_{g}, &\text{if s}=B2\\ l_{u} + \beta_{0}, &\text{if s}=N \end{array}\right.  $$

Here, *l*_*u*_ models the library size and is estimated median of the mean coverage of all gene. The variable *β*_*G*+1_ models the genome-wide average enrichment of CLIP-signal over backgrounds in peaks and is constrained to be positive, *β*_*g*_ models the gene expression and *β*_0_ models read abundance in regions with little coverage (e.g. intergenic or intronic regions).

We model the coverage in the background libraries in a similar way. Here, the expected coverage $X^{v}_{i}$ in the background-library *v* for each position *i* in a gene *g* is determined by:

3 $$ \log(X^{v}_{i}) = \left\{\begin{array}{ll} l_{v} + \beta_{g} - \beta_{G+1}, &\text{if s}=P\\ l_{v} + \beta_{g} + \beta_{G+1}, &\text{if s}=B1\\ l_{v} + \beta_{g}, &\text{if s}=B2\\ l_{v} + \beta_{0}, &\text{if s}=N \end{array}\right.  $$

For the GLM, we further assume that the mean-variance relationship of the negative binomial distribution is described by:

4 $$ \sigma^{2}(\mu) = \mu + c\mu^{2}, c\geq0  $$

Estimation of the parameters is performed by alternately estimating the GLM-parameters *β*=(*β*_0_,*β*_1_,…,*β*_*G*_,*β*_*G*+1_) and the over-dispersion parameter *c* using maximum-likelihood estimation. In order to ensure an equally good fit of the GLM for all states of the model, we weight the observations in each state by the inverse of the total number of observations in the state. Estimation of the GLM-parameters *β* is performed using iteratively reweighted least-squares (IRLS) [33]. In order to speed up the computation and make the solution computable in memory, we derived an implementation of IRLS, where all relevant components are sparse. To his end, we formulated the design matrix of the GLM such that the weighted pseudo-inverse has a sparse LU-factorization during parameter updating. This factorization in turn can be used to solve for the updated parameters. Thereby, we can circumvent the computation of the pseudo-inverse, which is in general non-sparse and costly to compute. Furthermore, this speeds up fitting of the GLM by orders of magnitude compared to standard implementations.

Using the fitted model, we can compute the probability of $X_{i}^{w}$ in library *w* and state *s* as $p(X_{i}^{w} | s) = \mathcal {NB}(\mu _{s}, \sigma ^{2}(\mu _{s}))$, where *μ*_*s*_ is derived using the GLM parameters. The joint probability of the coverage at position *i* in all libraries *p*(*X*_*i*_) is then given by:

5 $$ p(X_{i}) = \prod_{u\in U} p(X^{u}_{i}) \, \prod_{v\in V} p(X^{v}_{i})  $$

Modelling the coverage jointly across libraries allows accounting for the effect of local biases that affect the CLIP as well as the background library.

To improve convergence of the estimated GLM parameter for the background state *β*_0_, we set the gene expression parameter *β*_*g*_ in the computation of the emission probabilities such that all states have a higher expression rate than the background state. This is achieved by setting *β*_*g*_=*β*_0_+*β*_*G*+1_+10^−5^ if *β*_*g*_ is smaller than *β*_0_. Adjusting these parameters is typically only in the initial iterations and only for genes with few reads necessary.

### Diagnostic event model

To model diagnostic events and sequencing errors, we assume that peaks are a mixture of several classes of positions that have distinct rates of diagnostic events. In our model, we have found that 10 classes are typically enough. For each of the classes, we model the counts using a Multinomial-Dirichlet hierarchical model. In this model, the diagnostic events in all replicates at a given position are assumed to be distributed according to a multinomial distribution with parameter *q*. Here *q* models the rate of diagnostic events. This parameter is at each position identical in all replicates. To allow variation in the rates between positions in the same class as well as for excess variance, we model *q* to be drawn from a Dirichlet distribution with parameter *α*. The resulting model is described in the following. Denote by $N^{i}_{u}$ the number of reads covering a position *i* in replicate *u*∈{1,…,*U*} of the CLIP-libraries. Denote furthermore by $Y^{i,u}_{1},\dots,Y^{i,u}_{M}$ the number of occurrences for each of the *M* diagnostic events (all possible conversions, deletions of all bases and reads ends) in the reads at position *i* in replicate *u*. If we define $Y^{u}_{i}=(Y^{i,u}_{1},\dots,Y^{i,u}_{M}, N^{i}_{u}-\sum _{i=m}^{M} Y^{i,u}_{m})$, then the probability of observing *p*(*Y*^1^,…,*Y*^*U*^) in state *s* is given by:

6 $$ {}p(Y^{1}_{i},\dots,Y^{U}_{i}|s) =\sum_{s=1}^{10} \mu_{s} \int_{p} (\prod_{u=1}^{U}\mathcal{M}(Y^{u}_{j}|q))\mathcal{D}(q|\alpha^{s})\,dq,  $$

where the parameters $\alpha ^{s}\in \mathbb {R}^{M+1}$ and $\mathcal {M}$ and $\mathcal {D}$ denote the multinomial and Dirichlet distribution, respectively. For brevity, we denote $p(Y^{1}_{i},\dots,Y^{U}_{i})$ with *p*(*Y*_*i*_) in the remainder of the text. The parameters for the diagnostic event model are learned by maximizing the likelihood. Parameters for the peak state are fitted on the foreground dataset on the peak positions whereas parameters for the background states are fitted on the background dataset on the peak positions. Positions that are in regions where two or more genes overlap are ignored for learning the diagnostic event parameters, as diagnostic events are strand specific and overlapping genes on the opposite strand could dilute the learned signal. To speed up the fitting, we estimate the parameter on a subset of 1,000,000 randomly sampled positions with coverage. Furthermore, to increase the stability of the fitting, we use four random initializations from a uniform distribution and the solution of the previous iteration at each iteration of the EM-algorithm.

Finally, the emission probability of a state *s*∈{*P*,*B*1,*B*2,*N*} in the NHMM at a position *i* is given by the product of the probability for the coverage *p*(*X*_*i*_|*s*) and the observed diagnostic events *p*(*Y*_*i*_|*s*).

### Read filtering

To make the modelling of diagnostic events more robust, we discarded reads that had more than two mismatches. We also only consider reads that map to the same strand as the gene under consideration, if read strand information is available. In order to prevent mis-mapping read-ends from diluting diagnostic event profile estimation, we ignore conversions that occur in the first or last two bases of a read. Furthermore, we mask positions that are likely to be SNPs for diagnostic event modelling. To this end, we use information from the background dataset to determine whether a position has a SNP. For positions to be called a SNP, we require that they have at least 20 reads and that at least 20% have a conversion event in the background.

### Peak calling

Peaks are called by computing consecutive regions for which the peak state is the most-likely state in the NHMM using the Viterbi algorithm [34]. For computation of eCLIP peaks, we added during determination of the peak regions a penalty of −5 to the peak state in order to only predict the central high-confidence parts of peaks. The scores for a peak are computed as the log-likelihood ratio of the peak state versus the other states in NHMM at the peak location. P-values for a peak are computed in the following way. We first compute for each position of peak the expected total coverage and variance of the CLIP-reads. For this, we sum the expected mean and variance at each position of the peak. We then compute based on the cumulative distribution function of a negative binomial with the computed mean and variance, the p-value of the observed total coverage of the CLIP-reads. For our analyses we only consider peaks that have Bonferroni corrected p-value ≤0.05.

### Random controls for peak scores

To quantify motif enrichments that are due to chance, we randomly shuffled the peaks in the genes and computed for each random peak region the maximal motif score.

### Model fitting

We fit the parameters of the model using the EM-algorithm. Specifically, we iterate between estimating the parameters of the diagnostic event model, the expression modelling and the NHMM. For the analyses, this is done for at least 5 iterations. The model was run until full convergence was reached. As we observed that the parameters only changed minimally after 10 iterations, we stopped the model fitting after 10 iterations in order to speed up the data processing.

### Masking of miRNA genes

As a default option, we treat positions in genes that overlap annotated microRNA genes as if they had no coverage or diagnostic events.

### Motif discovery

For motif discovery we used RCAS [35] with the default parameters.

### Data acquisition

PAR-CLIP data for PUM2 was downloaded from SRA (SRP002487) [9]. eCLIP, shRNA-seq and RNA-seq data for the eCLIP analysis were downloaded from the ENCODE website (https://www.encodeproject.org) [13], HITS-CLIP data was obtained from SRA (SRP070745) [27], Ribo-zero data for HEK293 was obtained from SRA(SRP080811) [7] and iCLIP-data was obtained from ArrayExpress (E-MTAB-1371) [30]. The RBFOX2 position weight matrix (PWM) was obtained from [13] and the PUM2-PWM from [32]. Motifs for the eCLIP analysis were obtained from [26].

### Read processing

Reads for PAR-CLIP analyses were processed using PARpipe (Available from https://github.com/ohlerlab/PARpipe). Reads and quantification (e.g. site calls) for ENCODE eCLIP and shRNA-seq data were obtained from the ENCODE website (https://www.encodeproject.org). HITS-CLIP reads were quality-filtered using the fastx toolkit with the parameters -q 10 -p 95 [36] and trimmed adapters using cutadapt [37] with the parameters –overlap=3 -m 24 discarding untrimmed reads. Subsequently, reads were converted to fasta format and collapsed still including the four randomized nucleotides at both end of the reads. Randomized adapter ends got trimmed after read collapsing and added to the read identifier and treated as unique molecular identifiers (UMIs). Reads for the HITS-CLIP dataset were aligned using STAR (v.2.4.2a) [38]. Reads were first aligned and removed against the rRNA genome parts using the following parameters for *D.melanogaster*: –alignEndsType EndToEnd –outFilterMultimapNmax 10 -outFilterIntronMotifs RemoveNoncanonical –outReadsUnmapped Fastx –alignSJoverhangMin 12 –outFilterMatchNmin 15 –outFilterMismatchNmax 1 –outFilterMismatchNoverLmax 0.05 –outFilterMultimapScoreRange 3 –alignIntronMax 20000 –seedMultimapNmax 200000 –seedPerReadNmax 30000.The reads that did not align to the rRNA were then aligned against the *D. melanogaster* genome BDGP6 (Ensembl v81) using STAR with the following parameters: –alignEndsType EndToEnd –outFilterMultimapNmax 10 –outFilterIntronMotifs RemoveNoncanonical –alignSJoverhangMin 12 –outFilterMatchNmin 15 –outFilterMismatchNmax 1 –outFilterMismatchNoverLmax 0.05 –outFilterMultimapScoreRange 3 –alignIntronMax 20000 –seedMultimapNmax 200000 –seedPerReadNmax 30000 Reads with mismatches within the first and last two nucleotides were filtered out. Next, we removed reads with mismatches relative to the genome reference, which were likely introduced during sequencing and thus represent sequencing errors and not diagnostic events. To this end, we grouped alignments based on genomic coordinates (Chr, start, end, strand) and their UMIs. In case alignments overlapped entirely and shared the same UMI, while differing from each other and/or the reference sequence, we sorted by copy number (retained from read collapsing) and removed reads with relative lower copy number and a hamming distance one to the higher copy number reference read. For alignment of RNA-seq reads to the *human* genome, reads were aligned against the *human* genome GRCh37 using STAR with the following parameters: –alignEndsType EndToEnd –chimSegmentMin 40 –chimJunctionOverhangMin 40 –outFilterMultimapNmax 2–outFilterIntronMotifs RemoveNoncanonical –alignSJoverhangMin 16 –outFilterMatchNmin 30 –outFilterMismatchNmax 2–outFilterMultimapScoreRange 0 –alignIntronMax 20000 PAR-CLIP reads for PUM2 were aligned against the *human* genome GRCh37 using Bowtie [39] with the following parameters:-v 1 -m 10 –all –best –strata -p 4 -S iCLIP reads were aligned to *human* genome GRCh37 using STAR with the same parameters as the HITS-CLIP reads. Removal of PCR-duplicates was performed using UMI-tools [40].

To remove reads mapping to multiple locations in our analysis, we only kept the best alignment of a read if the second best alignment had more than one mismatch more than the best alignment. Furthermore, we discarded reads that had more than two mismatches.

### Application of methods for PAR-CLIP analysis

We called peaks with PARalyzer (v1.5), WavCluster (downloaded from https://github.com/FedericoComoglio/wavClusteR), Piranha(v.1.2.1) and BMIX (downloaded from https://github.com/cbg-ethz/BMix) using default parameters. For PAR-CLIP, peak calling with Piranha data yielded less than 10 peaks. Thus, we applied it without using a background dataset.

### Motif prediction

We predict motifs using biopython [41] using the pssm scoring scheme. For the motif calling a threshold score of 3.0 was used and only the forward strand was considered. Additionally a small pseudo count of 5∗10^−5^ was added to remove potential zeros in the PWM.

### De novo motif discovery and visualization

For de novo motiv discovery all peaks (*n*=29556) that can be annotated by mature mRNA annotation categories (3’utr, 3’utr-intron, 5’utr, 5’utr-coding, 5’utr-intron, coding, coding-3’utr,coding-5’utr, coding-intron, intron-3’utr, intron-5’utr, intron-coding, start-codon, stop-codon) were selected. For this analysis, the expressed transcripts per gene with highest RSEM isoform percentage from two total RNA-seq experiments in *Drosophila* S2 cells (personal communications Hans-Hermann Wessels) were selected. Subsequently HOMER2 (v.4.9.1) [42] was applied for de novo discovery using dinucleotide shuffled background sequences. For HOMER2 the following parameters were used: len 6 -strand + -p 4. The shuffled background was generated using uShuffle (v.0.2) [43] using the following parameters: -k 2 -n 10 -r 10004. To plot the motif position relative to peak summits, we used the Bioconductor package GenomicRanges (v.1.22.4) [44] to center in a + - 50nt window around the peak summit and searched for the motif PWM using the patternMatrix function from Genomation (v.1.2.2) [45] using the following parameters min.score=0.8, prior.params = c(A=0.25, C=0.25, G=0.25, T=0.25). To obtain a suitable background, we shuffled the PWM posterior probability from the retrieved GGAGGA motif for each nucleotide position randomly, but left the individual values unchanged to keep the overall PWM positional preference.

### Scoring for gene-based analyses

To combine peaks in for a gene we proceeded as follows. For omniCLIP we summed the scores. For Clipper and Piranha we summed the log p-values from peaks in both replicates for each gene.

### Data simulation

For simulating reads for a CLIP-seq experiment as well as a matching background, we proceeded as follows. First we drew the base gene expressions *g*_*i*_ for each gene *i* on chromosome 1 from the distribution $\exp (\mathcal {N}(10,4))$. Next, we chose for each gene randomly a representative isoform and sampled its expression for six replicates from a Negative binomial distribution with mean *g*_*i*_ and variance $g_{i} + 0.1\times g_{i}^{2}$. We then used the sampled transcript abundances to simulate reads using Flux-Simulator (v.1.2.1) [46]. This resulted in six libraries, each having ∼15×10^6^ reads. The first two libraries were used as background libraries. The next two libraries were used to simulate CLIP-seq data. To this end, we only kept reads that overlapped predicted PUM2 motifs and introduced a A-T conversion at the fourth position of the motif in 50% of the reads. To mimic the effect of stronger CLIP-signal for motifs that have a higher motif score, we sampled of each read a value *p* uniformly between the minimal and the maximal motif score Subsequently, we discarded the read if *p* was lower than the maximal motif score in the read. To simulate non-specific binding in the CLIP-libraries we added 10% from the remaining two libraries to the CLIP-seq libraries. Finally, we aligned the reads with STAR, as described above.

### Software availability

The software for omniCLIP can be obtained from: https://github.com/philippdre/omniCLIP under the GNU GPL license (v3). The version of source code used in this manuscript has been deposited at: 10.5281/zenodo.1320207.

## Additional file

Additional file 1 Figures S1–S3, Table S1–S2 Supplementary material. (PDF 76 kb)
