# Supplementary material for: omniCLIP: probabilistic identification of protein-RNA interactions from CLIP-seq data
Source: Genome Biol. 2018 Nov 1;19:183. doi: 10.1186/s13059-018-1521-2 (PMC6211453; doi:10.1186/s13059-018-1521-2)
Supplement: Supplementary file 1 — Figures S1–S3, Table S1–S2 Supplementary material. (PDF 76 kb) [file 13059_2018_1521_MOESM1_ESM.pdf]

# Supplemental tables

| **Gene name** |
|---|
| FXR1 |
| IGF2BP1 |
| KHDRBS1 |
| LIN28B |
| PCBP2 |
| RBFOX2 |
| SF3B4 |
| SRSF1 |
| SRSF7 |
| TARDBP |
| TIA1 |
| U2AF2 |

Table S1: Genes used for eCLIP analsyis

| | | | | | | |
|---|---|---|---|---|---|---|
| A | 0.001 | 0.001 | 0.902 | 0.001 | 0.103 | 0.997 |
| C | 0.240 | 0.001 | 0.001 | 0.072 | 0.085 | 0.001 |
| G | 0.758 | 0.997 | 0.001 | 0.926 | 0.811 | 0.001 |
| T | 0.001 | 0.001 | 0.096 | 0.001 | 0.001 | 0.001 |

Table S2: Motif that was found using HOMER for CG3800

# Supplemental figures

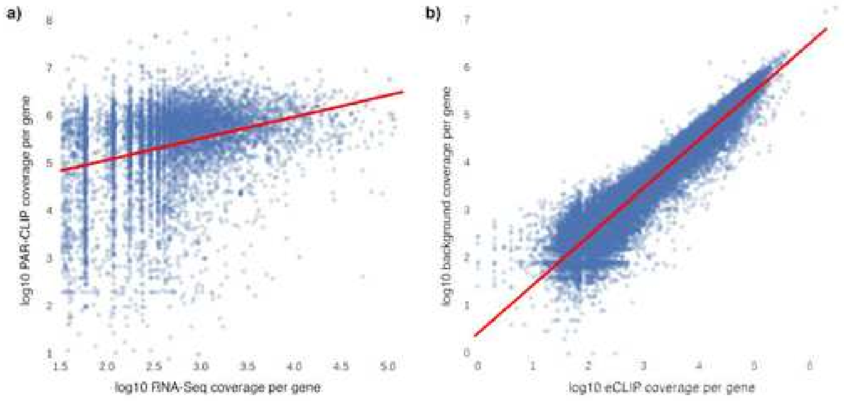


Figure S1: **Correlation of CLIP-Seq and background signal for PUM2** Shown in **(a)** is the summed coverage per gene in two PUM2 PAR-CLIP replicates and in two RNA-Seq replicates in HEK293 cells. Shown in red is the linear fit of the data. Shown in **(b)** is the summed coverage per gene in two PUM2 eCLIP replicates and their corresponding background library in K562 cells. Shown in red is the linear fit of the data.

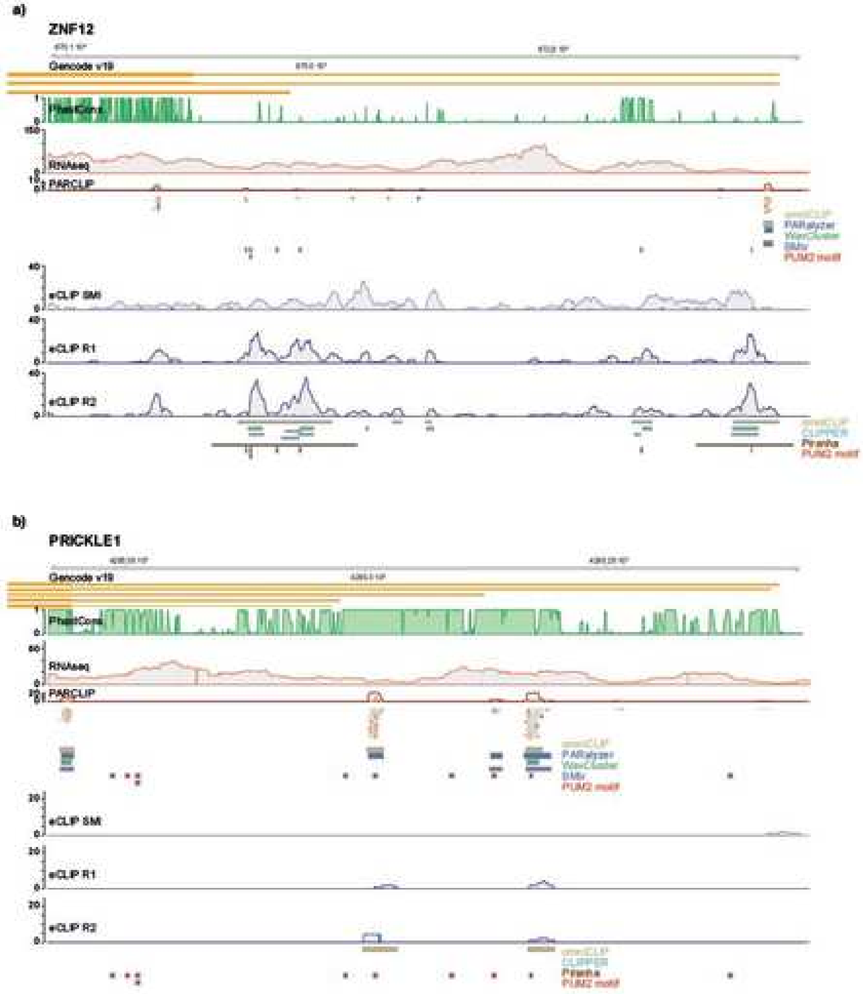


Figure S2: **Examples of peak finding across protocols.** Peak calling for the two transcripts ZNF12 and PRICKLE1 for PUM2 eCLIP libraries with a matched input from K562 cells, and for PUM2 PAR-CLIP libraries from HEK293 cells with an RNA-Seq background. On the top of each panel, Gencode v19 transcript isoforms are illustrated, as well as UCSC hg19 100way PhastCons conservation scores (green). Peak calls for omniCLIP (yellow), PARalyzer (black), WavCluster (green), BMix (purple), Clipper(turquoise) and Piranha (brown) are shown below the coverage profiles. PUM2-motifs with score higher than 8.0 are shown under the peak calls (in red). **a** The 3'UTR of ZNF12. omniCLIP can be used to robustly calls peaks on PUM2 CLIP data to determine cell type specific binding events, despite the data being generated by different specialized CLIP protocols and in across cell types. For PAR-CLIP data, individual read alignments (grey bars) are depicted to illustrate PARCLIP specific T-C conversions (organge ticks) relative to the reference genome. Due to their low depth the two PAR-CLIP libraries have been merged for the visualization. omniCLIP does not call a peak for the leftmost read cluster. This shows that the accurate modelling of diagnostic events as well as incorporation of the local background enables a better distinction of true high-confidence from low-confidence binding sites. **b** The 3'UTR of PRICKLE1. omniCLIP calls true binding sites in regions with sparse data, highlighting the benefit of using replicate information and having a well calibrated gene expression model.

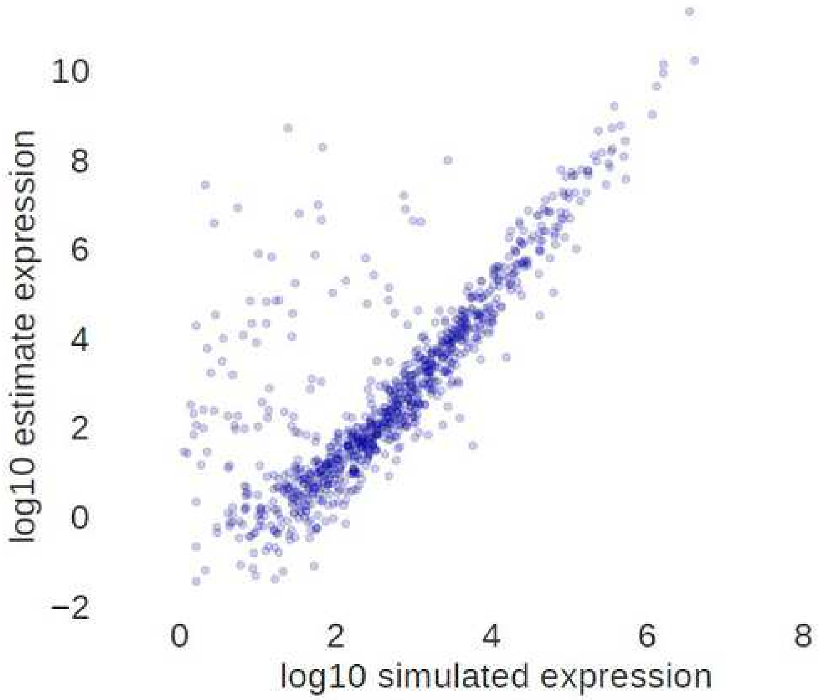


Figure S3: **Correlation of simulated and estimated gene expression.** Shown is the log10 estimated expression an the log10 simulated expression for each gene in the simulation.
